# Cognitive and neural plasticity in older adults’ prospective memory following training with the Virtual Week computer game

**DOI:** 10.3389/fnhum.2015.00592

**Published:** 2015-10-28

**Authors:** Nathan S. Rose, Peter G. Rendell, Alexandra Hering, Matthias Kliegel, Gavin M. Bidelman, Fergus I. M. Craik

**Affiliations:** ^1^Rotman Research Institute, BaycrestToronto, ON, Canada; ^2^School of Psychology, Australian Catholic University, MelbourneVIC, Australia; ^3^Faculté de Psychologie et des Sciences de l’Éducation, Université de GenèveGeneva, Switzerland; ^4^Institute for Intelligent Systems and School of Communication Sciences and Disorders, University of MemphisMemphis, TN, USA

**Keywords:** prospective memory, cognitive training, ERPs (event-related potentials), aging, activities of daily living (ADL), Virtual Week

## Abstract

Prospective memory (PM) – the ability to remember and successfully execute our intentions and planned activities – is critical for functional independence and declines with age, yet few studies have attempted to train PM in older adults. We developed a PM training program using the Virtual Week computer game. Trained participants played the game in 12, 1-h sessions over 1 month. Measures of neuropsychological functions, lab-based PM, event-related potentials (ERPs) during performance on a lab-based PM task, instrumental activities of daily living, and real-world PM were assessed before and after training. Performance was compared to both no-contact and active (music training) control groups. PM on the Virtual Week game dramatically improved following training relative to controls, suggesting PM plasticity is preserved in older adults. Relative to control participants, training did not produce reliable transfer to laboratory-based tasks, but was associated with a reduction of an ERP component (sustained negativity over occipito-parietal cortex) associated with processing PM cues, indicative of more automatic PM retrieval. Most importantly, training produced far transfer to real-world outcomes including improvements in performance on real-world PM and activities of daily living. Real-world gains were not observed in either control group. Our findings demonstrate that short-term training with the Virtual Week game produces cognitive and neural plasticity that may result in real-world benefits to supporting functional independence in older adulthood.

## Introduction

Prospective memory (PM) – the ability to remember and successfully execute our intentions and planned activities – is critical for successful, independent living in everyday life (Einstein and McDaniel, 1990; Ellis, 1996). PM failures account for between 50 and 80% of reported everyday memory problems (Crovitz and Daniel, 1984; Terry, 1988; Kliegel and Martin, 2003). The normal aging process has a substantial negative effect on PM performance (Henry et al., 2004; Bisiacchi et al., 2008; Kliegel et al., 2008; Rose et al., 2010; Ihle et al., 2013). As the world’s population ages, it is becoming increasingly important to develop ways to support successful PM functioning so that older adults can continue living independently, at home, without the need for assisted care. The present study attempted to train healthy older adults to perform everyday PM tasks using a computerized board game, called Virtual Week (Rendell and Craik, 2000). We aimed to assess if training gains could produce neuroplasticity in PM and transfer to improve real-world PM and everyday functioning. While previous attempts at using cognitive training to attain this goal have generally been unsuccessful (Reichman et al., 2010; Craik and Rose, 2012), the alternative method of “training for transfer” that we report here resulted in some encouraging results.

### Cognitive Training Approaches

Cognitive training programs have typically taken an approach that could be classified as either compensatory or restorative (Reichman et al., 2010). A compensatory approach attempts to teach an individual a specific strategy or technique that can be used to circumvent or compensate for a specific cognitive deficit (e.g., training participants to use a mnemonic strategy to facilitate memory encoding and/or retrieval, Verhaeghen et al., 1992; Gross et al., 2012). A restorative or process-based approach aims to repair or improve the functioning of neurocognitive processes that are involved more generally in many domains of cognition (e.g., adaptive working memory training, Morrison and Chein, 2011; Shipstead et al., 2012). Unfortunately, reliable evidence of far transfer of training gains to cognition in general or improvements in everyday functioning is sparse (Reichman et al., 2010; McDaniel and Bugg, 2012), which has led to the suggestion that training programs should be designed to “train for the transfer” (Craik and Rose, 2012). According to this view, if the desired outcome of training is to improve both PM in the real-world and competence in everyday living, then a training program should be designed to have participants practice performing real-world PM tasks in simulated everyday settings (Hering et al., 2014).

Despite the importance of PM in supporting everyday functioning (Kliegel and Bürki, 2012), few studies have attempted to train PM in older adults^1^. Some studies have taken a compensatory approach by training older adults to use the implementation intention strategy to encode prospective intentions more effectively and these studies have generally found benefits to real-world behaviors, such as participants’ ability to monitor their blood glucose levels (Liu and Park, 2004) or blood pressure levels (Brom et al., 2013; for a more thorough review, see Hering et al., 2014). Recently, one study has compared a compensatory with a restorative approach and found larger effects for a compensatory intervention (Brom and Kliegel, 2014). To date, no study has attempted to incorporate a train for transfer approach.

### The Virtual Week Training Study

**FIGURE 1:**
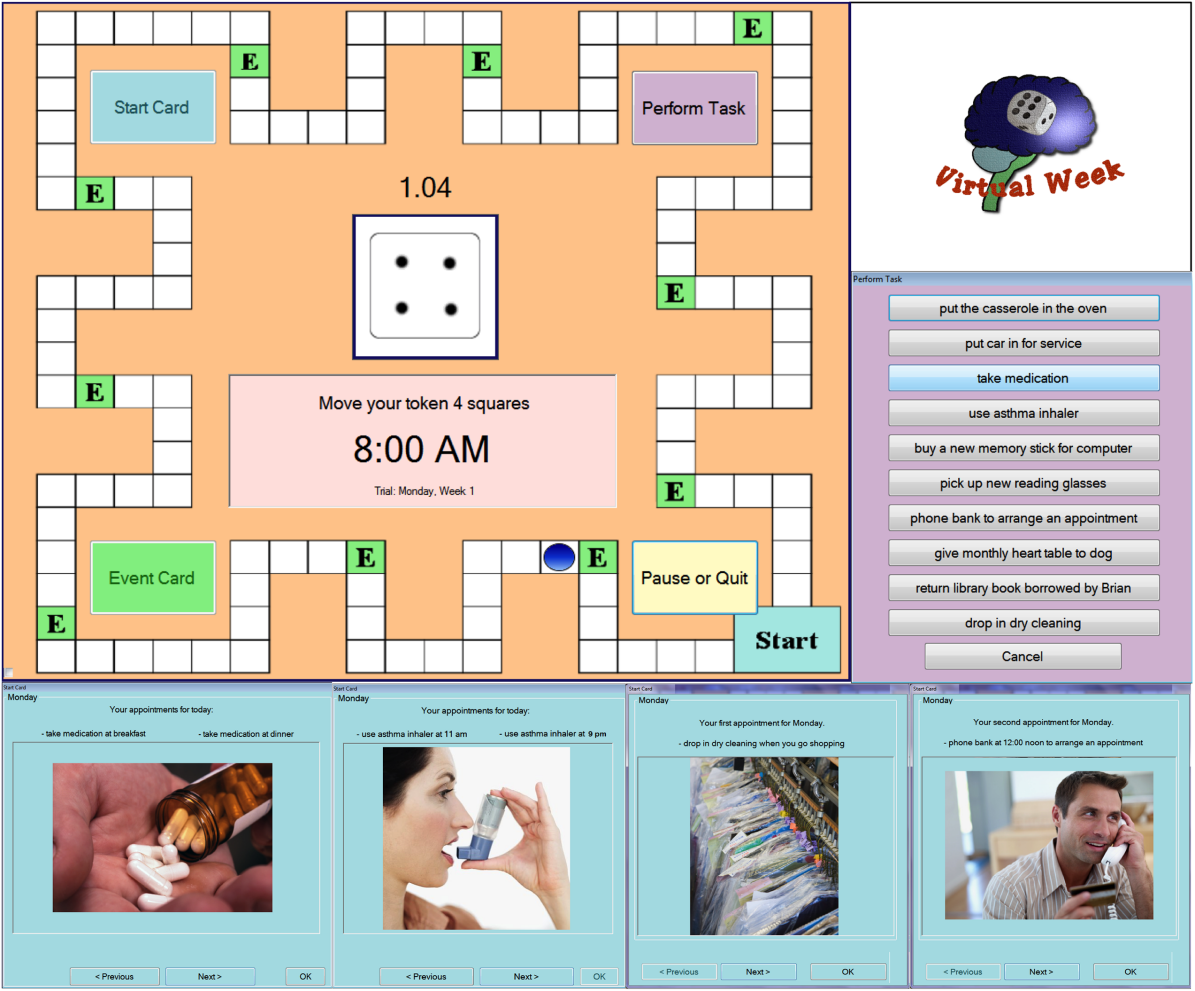
**Screenshots of the Virtual Week game board and examples of the perform task list and prospective memory tasks**.

The current study aimed to address three questions: (1) Can training improve older adults’ PM?; (2) Can training induce neural plasticity in brain mechanisms subserving PM?; and (3) Can training gains transfer to other tasks? To address these questions, we designed the Virtual Week training game. Virtual Week is a computerized game that simulates going through the course of a day on each circuit of the board, pretending to be engaged in events (e.g., choosing what to eat for meals or how to interact with others during events), and remembering to perform intended actions at the appropriate times (e.g., take medication at breakfast, deliver message to colleagues). **Figure 1** illustrates a screenshot of the board and examples of PM task instruction cards and the “perform task” list that contains tasks that the user is to perform.

Participants in the training group played 24 levels of the Virtual Week game over the course of approximately 1 month (three sessions/week, two levels/session). The difficulty of the game increased over the course of training in an adaptive manner in terms of the number of tasks to be completed per day, the complexity of tasks, and/or interference with prior tasks. The difficulty was titrated to each individual’s level of performance on the previous day. This feature, along with ample feedback and messages of encouragement about an individual’s performance on each day, served to maintain both an appropriate level of difficulty and the participant’s interest over the course of the training. Additionally, at the end of each week of training, participants were queried about the strategies they used to help remember to perform the PM tasks.

The primary outcome measures to index far transfer of training to improvements in everyday functioning were performance on a newly developed real-world PM task, the *Call-Back Task*, and the timed instrumental activities of daily living (IADL) task (Owsley et al., 2002). The call-back task required participants to carry out PM tasks at home, in their daily life, and therefore provided a proxy for their everyday PM performance. The IADL task captures a person’s efficiency in performing everyday-like activities, such as managing finances and following medication instructions (Tucker-Drob, 2011). This measure is an established and ecologically valid neuropsychological assessment used to assess an individual’s ability to function independently (e.g., Owsley et al., 2002; Tucker-Drob, 2011). Performance on IADL tasks is correlated with performance on other neuropsychological constructs such as speed, executive functioning, and episodic memory (Tucker-Drob, 2011). There is also some evidence that PM performance is associated with IADL performance, albeit based only on a self-rated questionnaire (Woods et al., 2012).

Other outcome measures that were assessed included the breakfast task (a lab-based measure of planning and task management Craik and Bialystok, 2006; Rose et al., 2015), and electrophysiological markers (ERPs) of PM performance embedded in a 2-back working memory task (West and Bowry, 2005). ERPs allowed us to detect potential changes in brain function that relate to the training. Specifically, it is unknown whether training could modulate ERP components associated with either PM cue detection (such as the N300 over occipital-parietal sites), retrieval of a PM intention and/or switching to initiating the intended action (such as the P3 or late prospective positivity (PP) complex over frontocentral and parietal sites), or both. Thus, a novel aspect of the current study was the examination of training related changes in ERP components that underlie PM.

To assess the effectiveness of training PM to inducing neurocognitive plasticity and producing transfer to other functions, differences between performance pre- and post-training were compared to performance levels in both active and passive control groups. The active control group participated in a music training program; the passive control group was a no-contact control group (see **Table 1** for characteristics of the groups). Comparisons between groups allowed us to assess the specificity of any training-related behavioral/neural gains and rule out the possibility that such changes result merely from differences in arousal and/or practice effects.

**Table 1:** Participant characteristics.

Group	*n*	Age (range)	Education (years)	Telephone Interview for Cognitive Status
Virtual Week training	23	67.4 (61–78)	15.1 (2.3)	34.7 (1.7)
Active control (music lessons)	14	66.4 (60–79)	15.1 (2.8)	36.5 (2.0)
No-contact control	18	68.5 (60–77)	15.8 (2.0)	36.7 (2.2)

## Materials and Methods

### Participants

The current study enrolled 59 healthy older adults. One participant was excluded due to health problems that arose during the study. Age ranged from 60 to 79 years (mean = 67.4; *SD* = 4.77); mean years of education (mean = 15.40; *SD* = 2.35); mean average vocabulary score on a split-half version of the Shipley Institute of Living Scale (mean = 17.33; *SD* = 2.51; Shipley, 1940; Zachary, 1986).

Participants were only eligible for the study if they were native English speakers or were fluent in English and had normal or corrected-to-normal vision and hearing. Furthermore, participants were only included if they did not have a history of a neurological or major psychiatric disorder and were not taking any psychoactive medication (e.g., anti-depressive, anxiolytics). To screen for normal cognitive functions the TICS-M (Telephone Interview for the Cognitive Status – modified; de Jager et al., 2003) was assessed; a cut-off score for participation of ≥31 was used (maximum = 41, cut-off for dementia = 25, Desmond et al., 1994). In the present study, mean TICS score was 35.8 (*SD* = 2.1).

Participants were recruited from the pool of volunteers of Baycrest Centre for Geriatric Care, from the Research Volunteer database of the Rotman Research Institute at Baycrest, or from respondents to a local newspaper advertisement. All participants provided written, informed consent. The study was approved by the Research Ethics Board of the Rotman Research Institute at Baycrest Hospital. Participants received a remuneration of $10 per hour for participation.

### Training Intervention

#### Virtual Week Training Program

In the Virtual Week game, participants roll a die and move a token around the board with one circuit around the board representing 1 day in which each day begins at 7:00 am and finishes at 10:15 pm (**Figure 1**). The number rolled indicates the number of spaces to move the token with one space representing 7.5 min of the day. During each “day” (circuit of the board), participants had to simulate performing plausible daily events at appropriate times during the day (e.g., eating breakfast, visiting the library). These events were indicated by green squares on the board. When the participant’s token passed an event square, they had to click on an event card icon. This caused a green event card window to pop up that described the particular event to the participant. The participant was to select their preferred activity for that event from three options.

Over the course of each virtual day, participants also had to perform different types of PM tasks. Some tasks were instructed at the beginning of the game and were to be performed every “day” without repeated instruction (*regular tasks*). Other tasks were to be performed only once during the game at a specific time or when encountering an event that occurred on that particular day (*irregular tasks*). These tasks varied according to the PM cue that triggered when the task was to be performed. Some tasks had to be performed during a specific event indicated by an appropriate event card (*event-based tasks*); other tasks had to be performed at a specific time of the virtual day indicated by a virtual clock on the screen that was calibrated to the position of the token on the board (*time-based tasks*). To perform a PM task, the participant had to click on the “perform task” button and select the correct task (e.g., “take medication”) out of a list of all the PM tasks for that day and four distractor tasks. The distractor tasks were included in the perform task list as lures so that correct performance required recollection of the correct task to be performed in relation to the cue.

A third task type (*time-check tasks*) required the participant to monitor a stop watch clock presented in the middle of the screen that displayed the amount of real time (in minutes and seconds) that had elapsed since beginning that virtual day. When the stop watch reached a specific time (e.g., 2 min:0 s or 4 min:0 s) the participant was to pause and perform the time-check task (i.e., test lung capacity) at that exact time by selecting the appropriate task from the perform task list.

The Virtual Week training program was similar to the original, computerized version of the game (Rendell et al., 2007; Rose et al., 2010) except that the time-based tasks in this study required monitoring a clock that indicated the virtual time of day rather than having the times marked on squares of the board (similar to recent Virtual Week studies, e.g., Leitz et al., 2009; Rendell et al., 2011), and the task content varied over the 24 virtual days. Many of the PM tasks simulated actual PM tasks that a sample of older adults (mean age = 75.4 years) reported in a survey study (Penningroth and Scott, 2013). Importantly, task difficulty increased over the 24 virtual days. For example, the first day of training started out with just two regular tasks and two irregular tasks (one event-based and one time-based task each). Upon completing at least 70% of the tasks correctly, the participant could proceed to the next level of difficulty. If participants did not attain the 70% correct criterion, they were required to repeat the day and reach this criterion before progressing to the next level. The proportion of tasks completed correctly and the number of times the day was repeated was recorded for each level of complexity.

The level of difficulty increased over the course of the training program by increasing the overall number of tasks to be performed during the day, the complexity of the tasks to be performed, and/or the amount of interference from prior tasks present during the day. For example, one way in which the task complexity was manipulated was by hiding the day clock and/or the time-check clock. In order to check the time, the participant had to click on a button to briefly view the time and then maintain an internal representation of the time as the day progressed. One way in which the amount of interference from prior tasks was manipulated was by switching the times and events at which the regular health tasks were to be performed on each day. For example, the health tasks on level eight, *take diabetes medication at breakfast and dinner*, and *check blood sugar at 11 am and 9 pm*, switched on level nine to *take diabetes medication at 10 am and dinner*, and *check blood sugar at breakfast and 7 pm.* Resolving the interference from previously learned associations was designed to simulate the difficulties one faces in real life when a doctor changes one’s medication. For a full list of the tasks that were to be performed over the 24 levels, see Supplementary Table S1.

#### Active Control Group (Music Training, Moreno et al., 2011)

An adapted version of the computerized training program developed by Moreno et al. (2011) was used as an active control group for comparison. While this program may result in small improvements in vocabulary learning in preschool-aged children, the program was not expected to produce any benefits to PM or everyday functioning in older adults. Critically, however, the program did involve a similar amount of “time-on-task” and involved some similar aspects to the Virtual Week training program, such as interaction with an experimenter/trainer and group training sessions. The Music Training group consisted of non-musicians that engaged in a music training program in a classroom setting with a teacher. Training sessions were between 40 and 60 min each; participants completed a total of 20 sessions during the 4-weeks period. The program involved a combination of motor, perceptual, and cognitive tasks and consisted of teaching participants basic musical concepts such as rhythm, pitch, melody, and voice.

#### No Contact Control Group

An additional group of participants participated only in the pre-test and post-test sessions separated by 1 month to determine the baseline rate of change due to practice effects and/or the passage of time between the pre-test and post-test sessions.

### Measures

#### PM Measures

Prospective memory performance was assessed using a battery of different PM paradigms. The tasks varied in their degree of ecological validity according to the classification of Phillips et al. (2008).

##### Virtual Week (Rendell and Craik, 2000)

All participants first performed a trial day on the pre-test session to learn how to play the game and how to perform examples of the different kinds of PM tasks before playing three virtual days of the original, computerized *Virtual Week* game (Rendell et al., 2007; Rose et al., 2010) during the pre-test session. All participants also performed three unique virtual days on the post-test with unique tasks in unique contexts in order to assess changes in performance before and after the training intervention (i.e., near transfer). Each virtual day consisted of 10 PM tasks per day: two time-check tasks, four regular and four irregular tasks. Two of the regular and irregular tasks were time-based tasks; the others were event-based tasks. The proportion of correctly selected PM tasks was calculated to score performance.

##### The call-back task

To assess naturalistic PM performance in everyday life, we developed a novel PM task – *the call-back task*. Participants chose a 2-h time slot during which they would be at home and able to call the research institute back and deliver a message. During this time slot, when participants were at home in their daily life, an experimenter called them and delivered the task instructions. The participant was to call the experimenter back at specific times (e.g., exactly 15 and 40 min after hanging up) and leave a message with their initials on the answering machine; the answering machine logged the time of the call. The procedure was repeated after 1 h with new times to call back after hanging up (e.g., 20 and 35 min). They were told to remember on their own and to call at the exact time that was indicated. Participants were explicitly warned to avoid using any reminder or timer and all participants reported complying with the instructions. Note that the target call-back times were never set to canonical clock times (e.g., quarter to, half past, or on the hour). The target clock time (e.g., 5:37 pm) was not mentioned to the participant. Times were counterbalanced across participants between the pre- and post-test sessions. The experimenter recorded the target clock time after hanging up and the absolute deviation in minutes between the target clock time and the actual time of the message was calculated. Times that were completely missed were recorded as 60 min, i.e., slightly longer than the latest response (50 min.). The dependent measure was the total minutes off from the target times for all four calls. The individual values were then log-transformed to account for non-normal distributions.

##### N-back + PM cues

Following West and Bowry (2005) we administered a computerized 2-back task with letters as an ongoing task and specific colors as PM cues to assess PM within a standardized laboratory setting. Participants saw a series of colored uppercase letters, one letter at a time. Their task was to press a key marked “YES” whenever the letter on-screen matched the letter presented two letters back. When the letter on the screen was not the same as the letter two letters back, they were to press the key labeled “NO” on the keyboard. Participants first performed a practice series of 20 letters on the letter 2-back task and the experimenter provided feedback. They then performed a series of 50 trials on the 2-back task (labeled as 2-back only). The letters in the series were sampled from a set of 15 English consonants in a pseudorandom order so that targets (2-back matches) appeared with 17 target and 33 non-target trials. The letters were presented in red, green, yellow, or blue font. For the initial 2-back only block, participants were told the color was irrelevant.

Following the 2-back only block, participants were informed they had an additional PM task to perform. They were instructed to press the spacebar instead of the “YES” or “NO” button whenever they saw a letter presented in a particular color (e.g., blue). Participants were informed of the color cue prior to performing a block of 50 trials. There were five “PM cues” (i.e., 10% of trials), 15 target trials, and 30 non-target trials per block. Participants performed a brief practice series on the 2-back task with PM cues where feedback was provided. Participants then performed four blocks of 2-back trials with PM cues. Participants were informed of a different color for the PM cue prior to each block. Accuracy and reaction times were computed for PM hits, 2-back targets and 2-back non-targets in the four 2-back + PM blocks, as well as for 2-back targets and non-targets in the 2-back only block.

##### Breakfast task (Craik and Bialystok, 2006)

The Breakfast task is a computerized simulation of the elements involved in preparing and serving a breakfast. Collectively, the components of the task broadly measure two neuropsychological functions: planning and multi-task management (Rose et al., 2015). Participants were required to prepare five different foods with different cooking times (e.g., sausages = 4.5 min, eggs = 2 min). The major aim was to have all foods ready at the same moment without under- or over-cooking any of the foods. Therefore, participants had to plan the right order of foods to start and stop at the appropriate moment. To do so they had to monitor the cooking times of each food indicated by blue time bars. Furthermore, participants had to set a table of four with all cutleries as quickly as possible. To perform the task participants had to use the mouse and press different buttons on the screen. There were two levels of difficulty. In the first trial participants saw all five foods, cooking time bars and the table to set on one screen. The second trial displayed each food with its cooking time bar and the table on six different screens that could be accessed by pressing several buttons. Therefore, the second trial placed higher demands on planning and monitoring.

The computer task allows assessing several different measures of planning and task management. Analyses focused on the *discrepancy of cooking times* and the *average deviation of start times*. For each food, there was an ideal cooking time and the actual time the participant cooked the food. The discrepancy of cooking times was the absolute difference between these two times in seconds and then summed for all five foods. In the event that a participant failed to start or stop a food, the average discrepancy of the remaining foods was used for the sum. In order to succeed on the breakfast task the participant had to plan the correct order in which to cook the five foods and to appropriately coordinate the starting times of each food. The absolute deviation between the ideal time to start cooking each of the foods and the actual start times was calculated and then averaged.

##### The Prospective-Retrospective Memory Questionnaire (PRMQ)

To assess self-reported PM performance in daily life we administered the PRMQ (Crawford et al., 2003). The PRMQ consists of 16 questions about prospective and retrospective memory failures in everyday life situations. Participants evaluate the frequency of each type of memory error on a 5-point scale (1 = never; 5 = very often). A sum score was calculated for the prospective component.

#### Everyday Competence

In addition to the various PM tasks, we administered a standard neuropsychological assessment of everyday competence.

##### Timed instrumental activities of daily living (TIADL; Owsley et al., 2002)

A modified version of the timed instrumental activities of daily living (TIADL) assessment was administered. Participants were required to complete five tasks: looking for a telephone number in a phone book, count out a certain amount of change in coins, read ingredients on three cans of food, point out items on a shelf as if they were shopping in a store, and read the directions on two medicine bottles. An experimenter read the instructions for all five tasks at the beginning of the test to the participant. They were then given a summary of the tasks to review until they were ready before starting. They were encouraged to hold all the tasks in mind as they were completing them. Each time they needed to look at the instructions again during the task, 10 s were added to their total time to completion. The total amount of time to completion was recorded.

#### Neuropsychological Measures

In addition to the PM and everyday competence assessments, we also administered a battery of tasks to assess neuropsychological functions, primarily for the purpose of examining the relations among neuropsychological functions, PM, and everyday competence in another report (Hering et al., manuscript in preparation). Based on previous research (e.g., Reichman et al., 2010), far transfer of PM training to improvements in neuropsychological functioning in general was not expected. Nonetheless, for the sake of completeness, performance on the following measures is reported.

##### Processing speed

The digit-symbol-coding test is a subtest from the Wechsler Adult Intelligence Scale III (WAIS-III; Wechsler, 1997) to assess information processing speed. The participants had to copy symbols to corresponding numbers as fast as possible during 2 min. The combination of the numbers 1–9 and the nine specific symbols was given on the top of the testing sheet and visible during the task. Performance was scored as the total number of correct items.

##### Inhibition

Inhibition was assessed via the Stroop test (Stroop, 1935). First, participants had to read out loud as quickly as possible 36 words naming the colors red, green, blue, or yellow. Afterward they had to name as quickly as possible the color of 36 squares printed in red, green, blue, or yellow. The last task was to name the ink color of 36 color names (i.e., red, green, blue, or yellow) printed in an incongruent color of ink. The measure of inhibition was calculated as the difference in time between task 3 and 2 – that is, the interference cost. Smaller values indicate better inhibition.

##### Working memory

A computerized version of the Corsi blocks paradigm (Milner, 1971) was administered to assess working memory. Participants saw a gray grid of 36 squares on the computer screen. During each trial a certain number of these squares turned blue, one after another. Participants had to remember the blue squares and reproduce the sequence at the end of their presentation by clicking on the squares of the grid that had displayed a blue square. The number of squares on each trial ranged from 3 to 8 with two trials at each sequence length. There were two blocks: one to reproduce the sequences in the same forward order as presented and one to reproduce the sequences in backward order, beginning with the item presented last and proceeding in reverse order to the item presented first. Performance was scored as the total number of correctly recalled squares.

##### Fluid intelligence

Raven’s Standard Progressive Matrices (Raven et al., 1996) was used to assess fluid intelligence. Participants were presented with several patterns and were asked to choose which one of five pieces fills a missing piece in order to complete the pattern. Participants were asked to complete as many problems as possible (out of 29 total items) within 10 min. The patterns increased in difficulty. Performance was scored as the total number of correctly solved patterns completed within the allotted time.

#### ERP Methods

A subset of the participants performed the n-back + PM cues while we recorded their electroencephalogram (EEG) so that we could obtain neurophysiological markers of PM cue detection. Specifically, we investigated event-related potentials (ERPs) associated with presentation of a PM cue during task performance (West and Bowry, 2005). Thirteen participants in the Virtual Week training group and 23 participants in the control groups completed both the pre- and post-test EEG sessions. The EEG was recorded with a Biosemi ActiveTwo system from an array of 64 Ag/AgCl electrodes placed over the scalp at locations according to the 10–20 system (Jasper, 1958). Vertical and horizontal eye movements were recorded from electrodes placed just lateral (Lo1, Lo2) and inferior (Io1, Io2) the right and left orbits. During online recording, electrode offsets were kept below 10 mV, referenced to Cz, and digitized at a 512 Hz sampling rate.

Neuroelectric recordings were processed using the EEGLAB 10 (Delorme and Makeig, 2004) and ERPLAB 1 (Lopez-Calderon and Luck, 2014) toolboxes and custom scripts coded in MATLAB (MathWorks, Natick, MA, USA). Electrodes were re-referenced to the common average, channels exhibiting excessive noise were interpolated (spherical spline), traces were epoched -200 ms before to 1200 ms after the time-locking stimulus event, and then bandpass filtered (0.01–100 Hz; IIR Butterworth, filter order = 4). Epochs with peak-to-peak deflections >150 μV were rejected prior to averaging. ERPs were cleaned of eyeblink artifacts using independent components analysis (PCA option, Wallstrom et al., 2004). ERPs for each condition were baselined to the prestimulus interval, and averaged in the time-domain. ERPs were computed for correct PM cue trials, n-back target trials, and n-back non-target trials. Responses were bandpass filtered (0.1–30 Hz) for analysis (a 10 Hz lowpass filter was applied for visualization). Remaining channels that exhibited excessive noise (e.g., TP9, TP10, FT9, FT10) were excluded from analysis. Data for two of the training participants were unusable due to excessive artifact (eye-blinks, skin potentials); data for three of the control participants were unusable because there were too few trials for analysis (only three correct PM trials or zero correct n-back target trials).

To measure ERP components associated with PM, mean voltages for correct PM cue trials were computed for each subject for the following components: N300 (250–400 ms), P3 (350–600), PP mean (600–1100 ms) and area under the curve (AUC; 350–1100 ms, to encompass the P3 and the PP slow wave). To minimize the family wise error rate for hypothesis testing, the components were measured in electrode clusters that captured left frontal (AF3, F1, F3, F5, FC1, FC3), right frontal (AF4, F2, F4, F6, FC2, FC4), centro-parietal (C1, Cz, C2, CP1, CPz, CP2), and left occipito-parietal (O1, PO3, PO7, P5, P7, P9), and right occipito-parietal (O2, PO4, PO8, P6, P8, P10) regions of interest.

### Procedure

After performing the telephone interview to assess general cognitive status, eligible participants were invited to the pre-test session that lasted 2–3 h. After participants provided informed consent, tests were administered in the following order: Virtual Week, Breakfast task, Stroop test, 2-back task with PM, Corsi block, Raven’s Standard Progressive Matrices, Shipley Institute of Living Scale vocabulary, and TIADL. During the testing session there were multiple short breaks for participants to rest. The session finished with the PRMQ and the general instructions for the Call-back task. Participants chose time windows to perform the Call-back task at home later on that day or on the following day.

Approximately 1 month after the pre-test session (duration range = 4–6 weeks), participants returned to the lab to complete alternate forms of the same tests on the post-test session. Alternate forms were counterbalanced across participants between the pre and post-test sessions.

## Results

### Training Gains

**FIGURE 2:**
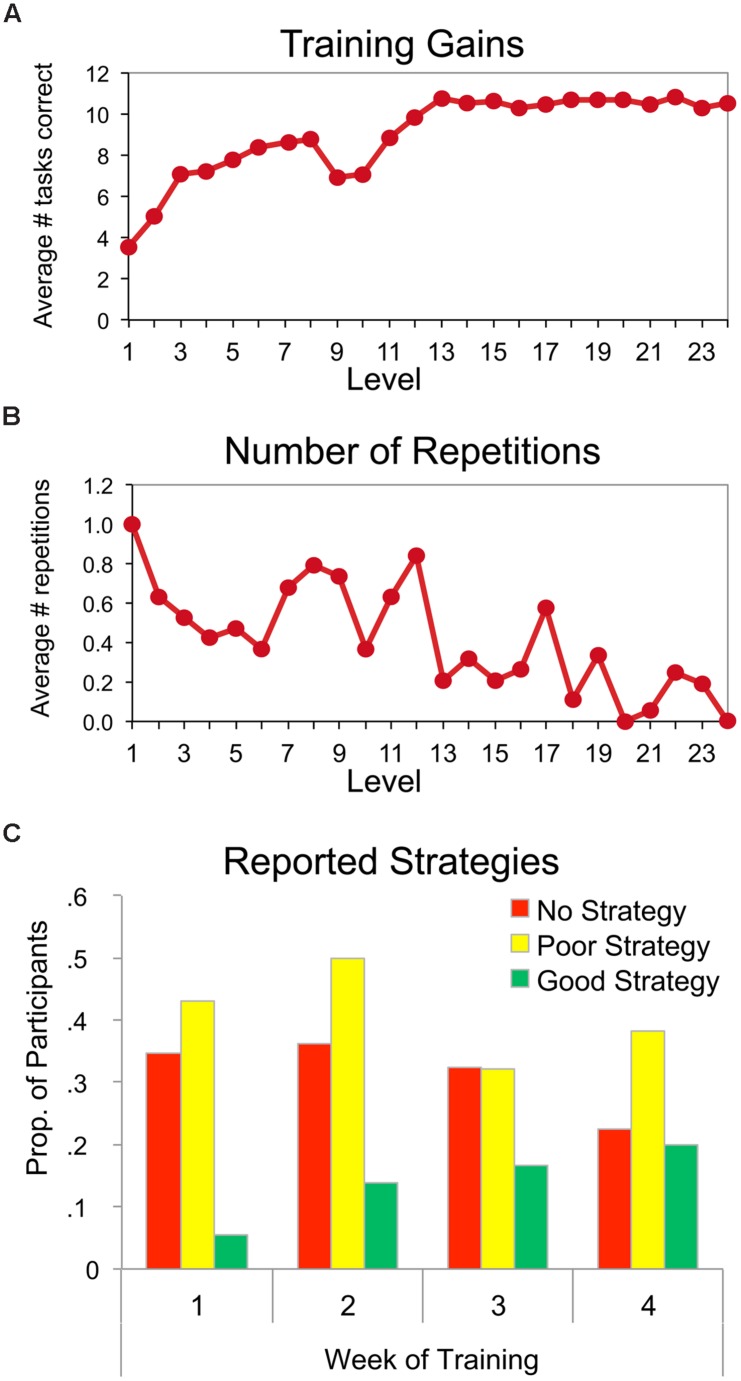
**(A)** Mean number of tasks correct and **(B)** mean number of repetitions as a function of the 24 levels of training and **(C)** distribution of reported strategies at the end of each week of training for the Virtual Week Training group.

We first assessed improvement in PM for participants in the Virtual Week group over the course of the training program. As can be seen in **Figure 2A**, the average number of PM tasks correctly performed on each virtual day of the training program steadily increased from an average of approximately 3.7 tasks at the beginning of training to approximately 10.5 tasks by the end of the training program. Obviously, the number of tasks that were administered on each day limited the maximum number of tasks that could be performed correctly on the day. Nonetheless, prior to training, participants could only remember to perform approximately 3–4 tasks correctly on the pre-test session; thus, the training gains represent more than a twofold increase in the capacity to perform PM tasks correctly. Additionally, **Figure 2B** shows the number of times a given day needed to be repeated before the criterion of 70% correct was obtained. The decrease in the number of times that each day needed to be repeated over the course of training is in spite of the increase in the difficulty and number of tasks that were to be performed on each day over the course of the training program.

**Figure 2C** illustrates the change in the strategies the training participants reported using to perform the PM tasks at the end of each week of training. Due to the variability in responses, the reported strategies were categorized into three classes: no strategy, poor strategies (i.e., rote rehearsal, ineffective/idiosyncratic strategy), or good strategies (i.e., visualization, association between cue and action). Three raters independently categorized each response, and collectively resolved the few discrepancies that existed. Good strategies were differentiated from poor strategies based on features of the responses that incorporated aspects of the implementation intentions strategy (Gollwitzer, 1999; Brom et al., 2013) and/or deep, elaborative encoding techniques (Craik and Lockhart, 1972) – strategies known to be effective in enhancing memory performance in prior research. In contrast, poor strategies contained features that were idiosyncratic or known to be less effective encoding techniques based on prior research (e.g., rote rehearsal; Craik and Lockhart, 1972). For example, one idiosyncratic strategy that was reported consisted of a participant trying to associate each to-be-performed PM task with one finger. The participant pressed the finger to the table while playing the game, which was to serve as a reminder for the task. As more PM tasks were introduced in the game, the participant tried to associate each new task with a new finger pressed to the table.

As can be seen in **Figure 2C**, the majority of strategies reported at the end of the first week of training represents ineffective strategies (no or poor). Over the course of training, the distribution of reported strategies shifted so that by the end of the fourth week, more responses were classified as good strategies. However, even after 4 weeks of training 3 days per week, the majority of responses were still classified as poor strategies. The potential for this limitation to have undermined the effectiveness of the training program is considered below in the discussion section.

### Training Induced Plasticity

We first compared the results of the active and control groups to determine if participating in the active “placebo” condition resulted in any training gains on the outcome measures. As predicted, there were no differences between the active (Music) and no-contact control groups on any of the outcome measures, all *p*s > 0.10 (Supplementary Table S2). Therefore, the active and no-contact control groups were combined and compared to the Virtual Week training group. One-tailed tests were used due to the directionality of the hypothesized benefits. This analysis allowed us to evaluate the potential of the Virtual Week training program to induce plasticity in the behavioral skills assessed before and after training.

**FIGURE 3:**
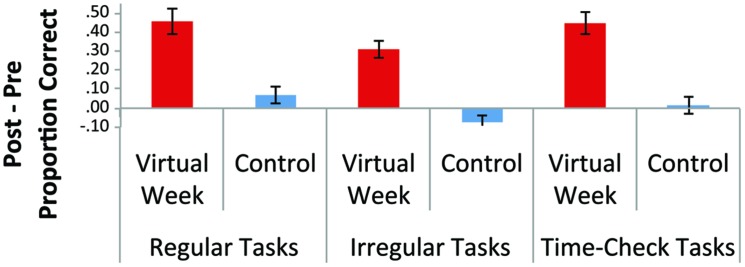
**Near transfer effects: improvements in the proportion of correctly performed prospective memory tasks on new virtual days**.

Mean performance on the pre and post-test for training and control groups on the outcome measures are presented in **Table 2**. We found large training-related gains in performance on all types of PM tasks on the unique virtual days for the training group relative to the control groups, which was confirmed by highly significant group by time-of-test (pre vs. post) interactions, *F*s > 30.0, *p*s < 0.001 (**Figure 3**). The task structure of Virtual Week was similar on the pre and post-tests and the training program and, therefore, training gains were to be expected. Nonetheless, the content of the virtual days and the PM tasks themselves differed, so the benefits of training were not specific to the particular tasks or task contexts learned during training (i.e., near transfer).

**Table 2:** Means on pre and post-test assessments for the training and control groups, and results of the group by time-of-test interaction effects.

	Training group		Control group		Group × Time of Test	
Measure	Pre	Post	Pre	Post	*F*	*p*
VW All Regular Tasks (%)	42	88	34	40	30.17	<0.001
VW All Irregular Tasks (%)	35	66	37	29	42.24	<0.001
VW Time-Check Tasks (%)	36	81	21	23	35.30	<0.001
Breakfast Dev. Cooking Time (sec)	198	155	299	262	0.03	ns
N-Back+PM Targets (%)	74	79	75	79	0.21	ns
N-Back+PM Hits (%)	54	64	52	58	0.03	ns
TIADLs (sec)	264	180	228	191	3.10	<0.05
Call-Back Task (min)	30.0	12.8	17.4	23.4	3.02	<0.05
PRMQ (PM rating)	9.7	9.6	12.0	13.6	0.71	ns

*VW, Virtual Week; TIADL, timed instrumental activities of daily living; PRMQ, Prospective-Retrospective Memory Questionnaire*.

**FIGURE 4:**
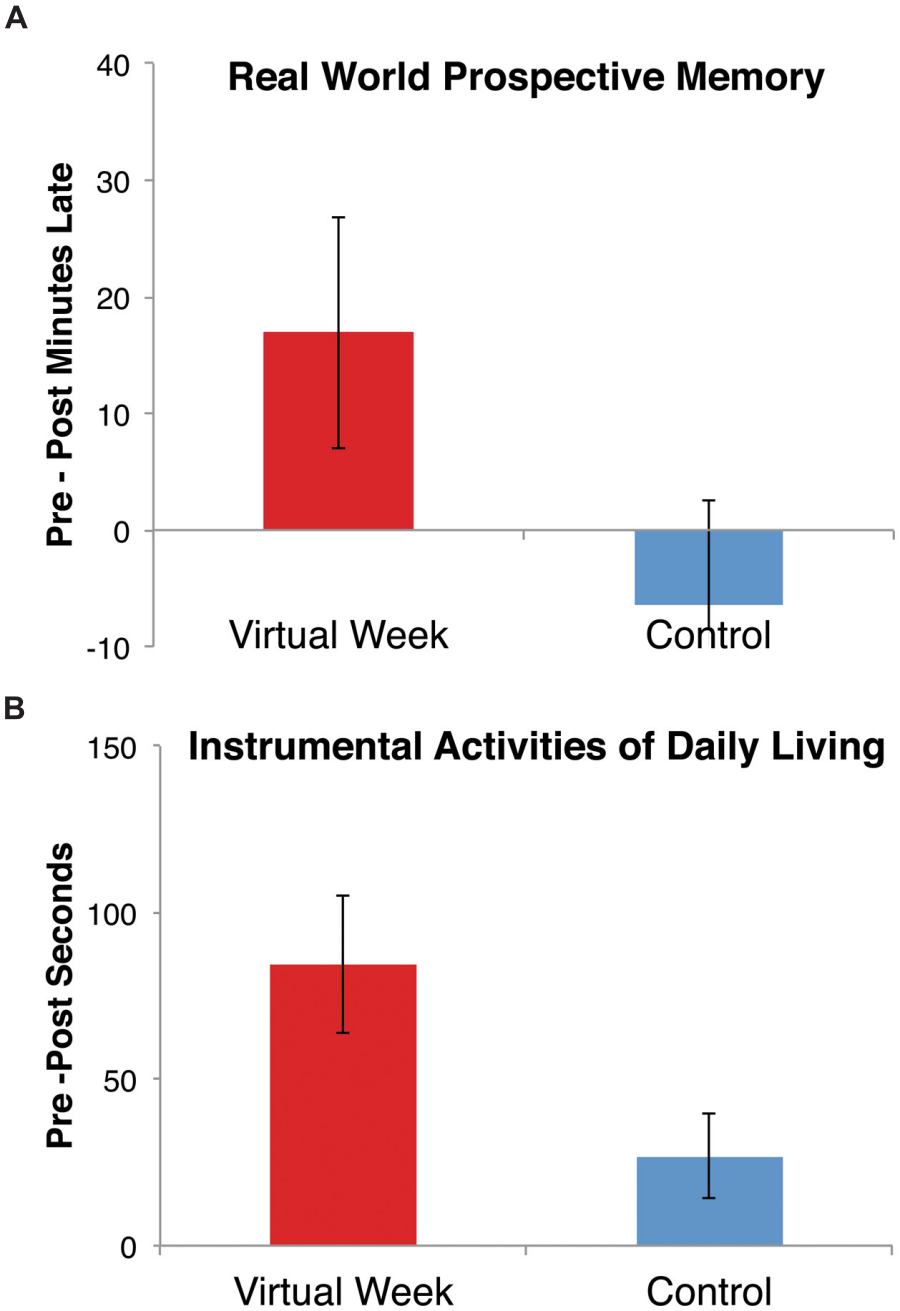
**Far transfer effects: reductions in **(A)** the number of minutes late on the real-world measure of prospective memory (the call-back task), and **(B)** the number of seconds to perform the instrumental activities of daily living tasks**.

No differences were observed between the groups in pre/post-test changes for the Breakfast task or laboratory PM measures. Although these tasks putatively measure aspects of PM, the lack of transfer is consistent with recent findings that suggest these measures more strongly relate to planning (in the case of the Breakfast task, Rose et al., 2015) and working memory (in the case of the n-back + PM cues, Hering et al., manuscript in preparation) than the PM processes tapped by the Virtual Week task. More importantly, both primary outcome measures – the call-back task (i.e., real-world PM) and the TIADL (i.e., functional independence) – showed significant transfer of training for the Virtual Week training group relative to the control group, *p*s < 0.05 (**Figure 4**). The significant reduction in the number of minutes late for the call-back task and the time to complete the instrumental activities of daily living following the Virtual Week training program is consistent with the notion of far transfer^2^.

### Neural Plasticity

**FIGURE 5:**
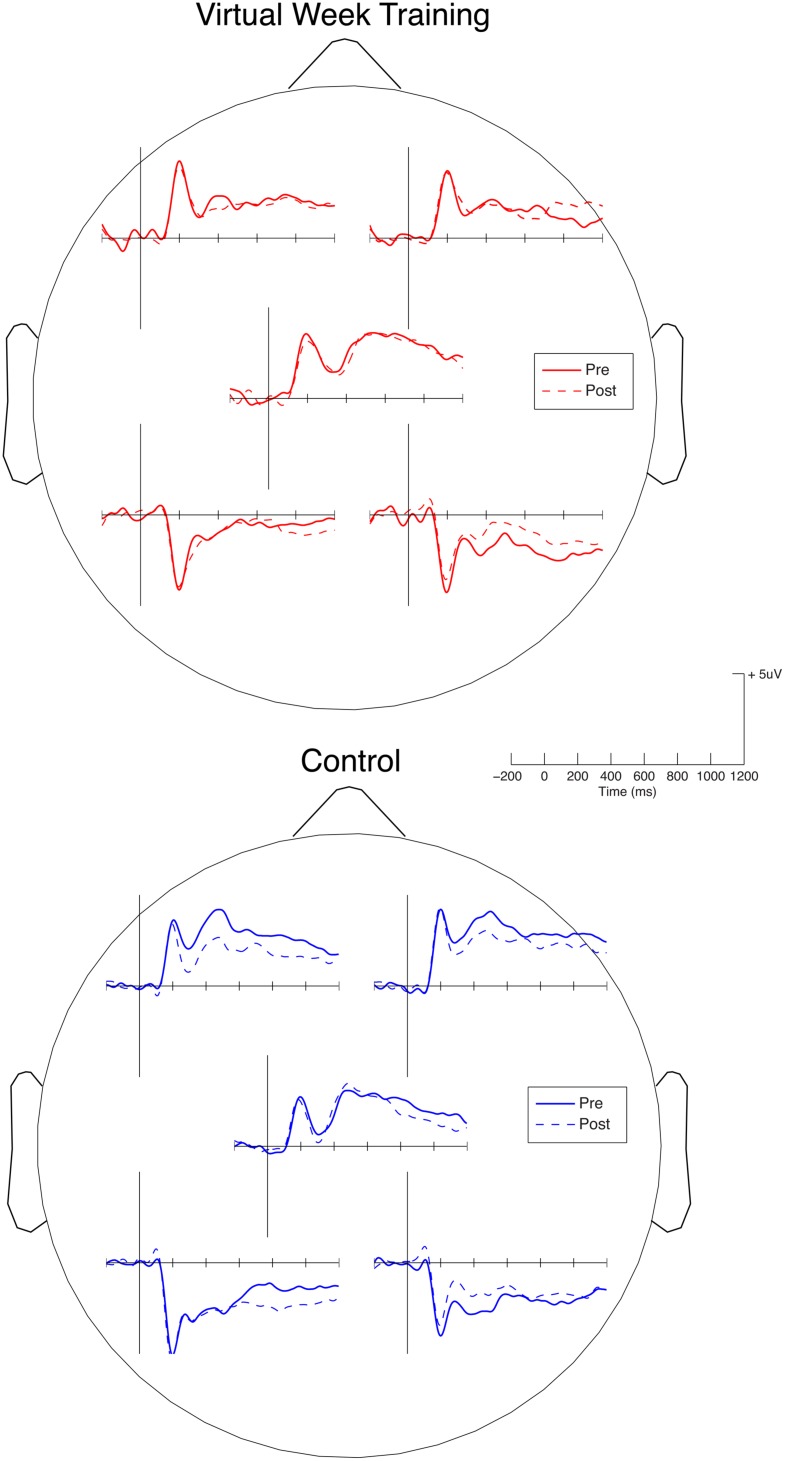
**Event-related potentials (ERPs) for correct PM trials for the Virtual Week Training group and the Control group on the pre and post-test in the left and right frontal, centro-parietal, and left and right occipito-parietal electrode regions of interest (see text for electrode cluster definitions)**.

To assess the potential for the Virtual Week training program to have produced neural plasticity, ERPs (N300, P3, PP) associated with PM cue detection on the pre and post-test sessions were compared for the training and control groups. The waveforms for the left and right frontal, centro-parietal, and left and right occipito-parietal region of interest clusters are presented in **Figure 5**. The only group × time-of-test interaction for the component measures in electrode clusters that reached significance was for the PP AUC measure for the right occipito-parietal cluster, *F*(1,31) = 9.15, *p* < 0.005, $\eta_{p}^{2}$ = 0.23. The waveforms were generally quite consistent between the pre and post-test sessions for both groups, with some decreases in timepoints between 300 and 1000 ms after stimulus onset, especially for the training group in the right occipito-parietal electrode cluster and for the control group in the frontal electrode clusters. There were increases in amplitude for the training group in the late positivity complex (∼900–1200 ms) in the right frontal electrode cluster and decreases in these timepoints in the left occipito-parietal cluster for both groups. However, with such small sample sizes providing analyzable data on both the pre and post-test, none of the effects in the frontal, centro-parietal, or left occipito-parietal clusters or correlations with behavioral performance survived appropriate thresholding for the family wise error rate. In sum, these preliminary ERP results provide some suggestion of neuroplasticity with training related reductions over right occipito-parietal cortex associated with correct PM performance, particularly in later stages during response selection and enactment.

## Discussion

Participants in the Virtual Week training program made substantial gains in PM performance (in the Virtual Week task) over the course of training, more than doubling the number of PM tasks that were performed correctly relative to control participants. Critically, this plasticity in PM functioning was not specific to the trained task, but also transferred to more efficient performance on a real-world measure of PM (the call-back task) and a neuropsychological measure of everyday competence (the TIADL). Some training related changes were also evident in neurophysiological markers of PM performance, despite a lack of concomitant behavioral effect in all participants in the Virtual Week training group.

Without observing behavioral gains that directly corresponded to the degree of modulation of ERPs pre and post-training, it is difficult to infer causation. Nonetheless, it is interesting to note that PM training was associated with more substantial modulation of later ERP components (500–1200 ms post-stimulus onset) that are thought to underlie processes associated with both PM retrieval and inhibiting or switching from performing an action associated with the ongoing task (e.g., pressing the match or non-match button for the n-back task with the right index or middle finger respectively) to performing the intended action associated with the PM cue instead (e.g., pressing the space bar with the left hand; for reviews, see West, 2011; Cona et al., 2015).

That training related modulations of the late positive complex were largely present in somewhat lateralized occipito-parietal and frontal sites over, for example, right inferior frontotemporal cortex may reflect the strong presence of competing intended actions for both the ongoing and PM response and the need to withhold a dominant (ongoing) response and engage a less frequent (albeit salient PM) response. Furthermore, both groups (especially the control group) showed reductions during times around the N300 and P3 components in left anterior frontal sites between the pre and post-test for correct PM responses, with the training group showing a significant decrease in the late PP complex in the right occipito-parietal region of interest. Such modulations are consistent with the notion of enhanced neural efficiency, which would be expected if performing the PM task was associated with more automatic retrieval of the intended action (Cona et al., 2015). Thus, the results provide some evidence of neuroplasticity, which may be seen as analogous to the differences in ERP components for PM tasks that involve more automatic/spontaneous retrieval than controlled monitoring, such as tasks with focal vs. non-focal cues (Cona et al., 2013). The overall pattern of ERPs and their modulation before and after training may reflect enhanced neural efficiency in brain regions associated with managing the competing demands for turning intentions into actions, consistent with more automatic/spontaneous retrieval and performance of the intended actions (Cona et al., 2015).

While these results are encouraging, they represent a first step in exploring the efficacy of PM training with the Virtual Week training program. The sample sizes in this preliminary study are rather small and a limitation in the recruitment procedures prevented a truly random allocation to conditions, which resulted in some small but potentially important differences between the groups. Nonetheless, our preliminary findings warrant further investigation in a larger, randomized controlled trial to replicate and extend the observed benefits of PM training to real-world PM performance and functional independence. It may also be noted that, while musical training may benefit older adults’ ability to process auditory cues and speech (Bidelman and Alain, 2015), the lack of a difference between this “active” control group and the no-contact control group suggests such benefits might be limited to specific trained tasks and/or stimuli rather than promoting far transfer. Future studies would benefit from exploiting the “train for transfer” principle that was incorporated into the Virtual Week training program and assessing whether training gains result in real-world benefits over time with a longitudinal study.

One potentially important limitation of the Virtual Week training program was evident in the strategies that participants reported using. At the beginning of training, most participants reported using either no strategy or a strategy that was considered to be ineffective (rote rehearsal). We had assumed that the Virtual Week training program – which was designed to be a restorative, process-based training program – would result in participants gradually learning to use more effective processes for encoding and retrieving their prospective intentions over the course of training. However, even after 4 weeks of three sessions per week, most participants still reported using ineffective strategies for prospective remembering by the end of the training program. Encouragingly, the overall distribution of participants shifted such that more participants reported using a strategy that PM researchers would consider to be an effective strategy. For example, by the end of the third week, the participant that reported an idiosyncratic strategy of associating each to-be-remembered PM task with a finger reported that the strategy did not appear to be a very good one and decided to try something different. Perhaps larger training and transfer gains could be attained if training had lasted longer or we had explicitly instructed participants to practice using more effective strategies as part of the training program.

There is ample evidence for large benefits to prospective remembering by teaching participants to use the implementation intentions encoding strategy (McFarland and Glisky, 2011; Brom et al., 2013; Brom and Kliegel, 2014; McDaniel et al., 2014). For example, McDaniel et al. (2014) incorporated implementation intentions strategy instructions as part of their extended, multi-domain training program and found that training resulted in large gains in PM performance on the Virtual Week game, their primary outcome measure. Additionally, in single-session strategy training experiments, teaching the implementations intention strategy to healthy older adults (Rendell et al., manuscript in preparation) and even individuals with early Alzheimer’s Disease (Lee et al., 2015; Shelton et al., submitted) showed substantial improvements in PM performance, such that the large age-deficit seen on event-based PM tasks was eliminated following implementation intentions strategy training (Rendell et al., manuscript in preparation). Such gains are not limited to performance on PM tasks performed in the laboratory. Brom et al. (2013), for example, showed gains in a real-world health behavior, blood-glucose monitoring, following implementation intentions strategy training.

Collectively, large, meaningful gains in PM functioning were observed following strategy training. However, it is unclear whether a compensatory approach to cognitive training would result in far transfer of training gains to untrained behaviors that are important for independent functioning in the real-world. For example, teaching individuals to use specific strategies are notoriously tied to the specific tasks in which they are practiced (McDaniel and Bugg, 2012), whereas self-generated processes learned in more restorative, process-based training programs may provide a more generalizable mode of processing that could be applied to varied contexts, including behaviors important for functional independence (Craik and Rose, 2012). The efficiency and generalizability of these two types of cognitive training are important issues to explore in future research.

## Conclusion

A short duration of cognitive training with the Virtual Week training program resulted in rather large training related gains in PM performance and some small changes in neural correlates of PM processing. Training gains resulted in far transfer in the form of enhanced accuracy and efficiency in performing real-world PM tasks and instrumental activities of daily living. The Virtual Week training program, which incorporates a “train for transfer” principle, represents an innovative avenue for cognitive training and potentially enhancing functional independence.

## Author Contributions

NR, PR, AH, MK, and FC designed the study. NR, PR, and AH helped develop the training program. NR and AH collected the data. NR and AH analyzed the behavioral data. NR and GB analyzed the ERP data. NR and AH wrote the paper with edits from PR, MK, GB, and FC.

## Conflict of Interest Statement

The authors declare that the research was conducted in the absence of any commercial or financial relationships that could be construed as a potential conflict of interest.

## Footnotes

1 There is a small literature on PM training in clinical samples (Raskin and Sohlberg, 1996; Fleming et al., 2005; Kinsella et al., 2009; Shum et al., 2011; Radford et al., 2012), but review of these is beyond the scope of the current study (for reviews, see Fish et al., 2010; Piras et al., 2011).

2 As expected, no differences were observed between the training and control groups on any of the background neuropsychological tests on either the pre or post-test, all *p*s > 0.05.
