# Supplementary material for: Cognitive and neural plasticity in older adults’ prospective memory following training with the Virtual Week computer game
Source: Front Hum Neurosci. 2015 Oct 28;9:592. doi: 10.3389/fnhum.2015.00592 (PMC4623669; doi:10.3389/fnhum.2015.00592)
Supplement: Supplementary file 1 [file Table_1.DOCX]

**Supplemental Table 1**

| Number | Day | Week | Level | Description |
| --- | --- | --- | --- | --- |
| 1 | Monday | 1 | 1 | medication @ breakfast & dinner, diabetes pill @ 11AM & 9PM |
| 2 | Tuesday | 1 | 2 | medication @ breakfast & dinner, diabetes pill @ 11AM & 9PM + 2 irregular |
| 3 | Wednesday | 1 | 3 | medication @ breakfast & dinner, diabetes pill @ 11AM & 9PM + 4 irregular |
| 4 | Thursday | 1 | 4 | medication @ breakfast & dinner, diabetes pill @ 11AM & 9PM + 4 irregular w/ HIDDEN CLOCK |
| 5 | Friday | 1 | 5 | medication @ breakfast & dinner, diabetes pill @ 11AM & 9PM + 4 irregular + 1 time-check (test blood sugar @ 2 minutes), |
| 6 | Monday | 2 | 6 | medication @ breakfast & dinner, diabetes pill @ 11AM & 9PM + 4 irregular + 2 time-check (test blood sugar @ 2 and 6 minutes) |
| 7 | Tuesday | 2 | 7 | medication @ breakfast & dinner, diabetes pill @ 11AM & 9PM + 4 irregular + 2 time-check (test blood sugar @ 2 and 6 minutes) w/ REPEATED LURES |
| 8 | Wednesday | 2 | 8 | medication @ breakfast & dinner, diabetes pill @ 11AM & 9PM + 4 irregular + 2 time-check (test blood sugar @ 2 and 6 minutes) w/ HIDDEN CLOCKS |
| 9 | Thursday | 2 | 9 | 4 NEW health tasks (e.g., medication @ 10 AM & lunch, diabetes pill @ breakfast & 8 PM) + 4 irregular |
| 10 | Friday | 2 | 10 | medication @ 10 AM & lunch, diabetes pill @ breakfast & 8 PM) + 4 irregular w/ REPEATED LURES |
| 11 | Monday | 3 | 11 | medication @ 10 AM & lunch, diabetes pill @ breakfast & 8 PM) + 4 irregular + 2 time-check (test blood sugar) @ 2 and 6 minutes |
| 12 | Tuesday | 3 | 12 | medication @ 10 AM & lunch, diabetes pill @ breakfast & 8 PM) + 4 irregular + 3 time-check (test blood sugar) @ 2, 4, and 6 minutes |
| 13 | Wednesday | 3 | 13 | medication @ 10 AM & lunch, diabetes pill @ breakfast & 8 PM) + 4 irregular + 4 time-check (test blood sugar) @ 2, 4, 6, and 8 minutes |
| 14 | Thursday | 3 | 14 | medication @ 10 AM & lunch, diabetes pill @ breakfast & 8 PM) + 4 irregular + 4 time-check (test blood sugar) @ 2, 4, 6, and 8 minutes w/ REPEATED LURES |
| 15 | Friday | 3 | 15 | medication @ 10 AM & lunch, diabetes pill @ breakfast & 8 PM) + 4 irregular + 4 time-check (test blood sugar) @ 2, 4, 6, and 8 minutes w/ HIDDEN CLOCKS |
| 16 | Monday | 4 | 16 | medication @ 10 AM & lunch, diabetes pill @ breakfast & 8 PM) + 4 irregular + 4 time-check (test blood sugar) @2, 4, 6, and 8 minutes w/ HIDDEN CLOCKS |
| 17 | Tuesday | 4 | 17 | medication @ 10 AM & lunch, diabetes pill @ breakfast & 8 PM) + 4 irregular + 2 time-check (test blood sugar) @ 2, 4, 6, and 8 minutes w/ HIDDEN CLOCKS w/ REPEATED LURES |
| 18 | Wednesday | 4 | 18 | medication @ 10 AM & lunch, diabetes pill @ breakfast & 8 PM) + 4 irregular + 2 time-check (test blood sugar) @2, 4, 6, and 8 minutes w/ HIDDEN CLOCKS w/ REPEATED LURES |
| 19 | Thursday | 4 | 19 | medication @ breakfast & dinner, diabetes pill @ 11AM & 9PM, test blood sugar @2, 4, 6, and 8 minutes |
| 20 | Friday | 4 | 20 | medication @ breakfast & dinner, diabetes pill @ 11AM & 9PM, test blood sugar @2, 4, 6, and 8 minutes w/ HIDDEN CLOCKS |
| 21 | Monday | 5 | 21 | medication @ breakfast & dinner, diabetes pill @ 11AM & 9PM, test blood sugar @2, 4, 6, and 8 minutes w/ HIDDEN CLOCKS w/ REPEATED LURES |
| 22 | Tuesday | 5 | 22 | medication @ breakfast & dinner, diabetes pill @ 11AM & 9PM, test blood sugar @ 2:15, 4:40, 6:30, & 8:20minutes |
| 23 | Wednesday | 5 | 23 | medication @ breakfast & dinner, diabetes pill @ 11AM & 9PM, test blood sugar @ 2:15, 4:40, 6:30, & 8:20 minutes w/ HIDDEN CLOCKS |
| 24 | Thursday | 5 | 24 | medication @ breakfast & dinner, diabetes pill @ 11AM & 9PM, test blood sugar @ 2:15, 4:40, 6:30, & 8:20 minutes w/ HIDDEN CLOCKS w/ REPEATED LURES |

**Supplemental Table 2.** Mean (SEM) scores on all tasks and measures on the pre- and post-test for the Virtual Week Training group, the Music Training group, and the No-Contact Control group.

|  | Virtual Week Training | | Music Training | | No-Contact Control | |
| --- | --- | --- | --- | --- | --- | --- |
| Task/Measure | Pre | Post | Pre | Post | Pre | Post |
| Digit Symbol Substitution (#) | 65.0 | 70.4 | 55.1 | 60.9 | 58.8 | 60.3 |
|  | 4.0 | 4.1 | 3.9 | 4.0 | 3.7 | 3.8 |
| Stroop Interference (s) | 20.6 | 19.4 | 24.1 | 20.9 | 20 | 17 |
|  | 2.5 | 1.8 | 2.4 | 2.4 | 1.9 | 1.2 |
| Stroop # Errors | 0.10 | 0.10 | 0.40 | 0.10 | 0.10 | 0.10 |
|  | 0.08 | 0.08 | 0.29 | 0.14 | 0.08 | 0.08 |
| N-Back Targets (%) | 63 | 60 | 72 | 73 | 64 | 63 |
|  | 06 | 08 | 03 | 03 | 06 | 07 |
| N-Back Targets RT (ms) | 834 | 840 | 828 | 813 | 838 | 811 |
|  | 31 | 38 | 30 | 29 | 23 | 38 |
| N-Back Non-Targets (%) | 79 | 86 | 74 | 76 | 69 | 78 |
|  | 03 | 02 | 05 | 05 | 05 | 04 |
| N-Back Non-Targets RT (ms) | 854 | 812 | 832 | 793 | 805 | 776 |
|  | 24 | 25 | 31 | 26 | 24 | 32 |
| Corsi Blocks (# correct) | 57.3 | 58.4 | 54.6 | 56.8 | 51 | 60.1 |
|  | 3.0 | 2.6 | 4.8 | 5.3 | 5.3 | 4.1 |
| Raven (#) | 17.0 | 17.4 | 14.6 | 15.3 | 15.8 | 17.4 |
|  | 0.8 | 0.8 | 1.5 | 1.3 | 1.2 | 0.9 |
| TIADL (s) | 264 | 180 | 252 | 217 | 209 | 170 |
|  | 34 | 18 | 27 | 25 | 26 | 13 |
| TIADL (# errors) | 11.3 | 10.5 | 10.2 | 10 | 9.7 | 9.9 |
|  | 0.7 | 0.6 | 0.4 | 0.4 | 0.3 | 0.4 |
| Call-Back Task (min) | 30.0 | 12.8 | 16.9 | 22.4 | 17.8 | 25.1 |
|  | 14.0 | 4.5 | 4.1 | 9.4 | 4.7 | 9.2 |
| PRMQ-PM | 9.4 | 9.4 | 14.3 | 15.1 | 10.2 | 12.5 |
|  | 0.8 | 0.7 | 0.9 | 0.8 | 1.4 | 0.8 |
| PRMQ-RM | 7.5 | 7.9 | 14.3 | 15.3 | 9.9 | 12.4 |
|  | 0.6 | 0.5 | 1.0 | 0.7 | 1.4 | 0.9 |
| PM+N-Back N-Back Targets (%) | 56 | 61 | 50 | 57 | 54 | 60 |
|  | 05 | 04 | 05 | 06 | 05 | 05 |
| PM+N-Back N-Back Targets RT (ms) | 885 | 847 | 855 | 855 | 825 | 870 |
|  | 30 | 20 | 41 | 42 | 39 | 23 |
| PM+N-Back N-Back Non-Targets (%) | 81 | 79 | 73 | 80 | 79 | 80 |
|  | 03 | 03 | 06 | 04 | 03 | 03 |
| PM+N-Back N-Back Non-Targets RT (ms) | 879 | 874 | 833 | 875 | 835 | 852 |
|  | 18 | 24 | 37 | 38 | 33 | 21 |
| PM+N-Back PM Targets (%) | 73 | 77 | 77 | 84 | 74 | 74 |
|  | 04 | 05 | 04 | 03 | 04 | 05 |
| PM+N-Back PM Targets RT (ms) | 842 | 852 | 837 | 794 | 813 | 818 |
|  | 26 | 29 | 13 | 30 | 29 | 23 |
| VW Regular Event Tasks (%) | 43 | 93 | 34 | 24 | 48 | 25 |
|  | 09 | 07 | 09 | 08 | 08 | 06 |
| VW Regular Time Tasks (%) | 41 | 78 | 19 | 23 | 38 | 22 |
|  | 08 | 05 | 06 | 08 | 07 | 05 |
| VW Time Check Tasks (%) | 37 | 83 | 11 | 23 | 30 | 22 |
|  | 07 | 09 | 05 | 10 | 07 | 09 |
| VW Irregular Event Tasks (%) | 50 | 69 | 38 | 16 | 38 | 22 |
|  | 06 | 07 | 08 | 04 | 06 | 05 |
| VW Irregular Time Tasks (%) | 16 | 55 | 19 | 29 | 32 | 38 |
|  | 07 | 07 | 06 | 08 | 06 | 06 |
| VW All Regular Tasks (%) | 42 | 88 | 29 | 44 | 43 | 54 |
|  | 04 | 05 | 07 | 05 | 07 | 04 |
| VW All Irregular Tasks (%) | 35 | 66 | 32 | 27 | 46 | 30 |
|  | 07 | 06 | 07 | 09 | 06 | 07 |

Note- RT = Reaction Time, TIADL = Timed Instrumental Activities of Daily Living, Prospective-Retrospective Memory Questionnaire, PM = Prospective Memory, RM = Retrospective Memory, VW = Virtual Week
